# 

**Published:** 1895-09

**Authors:** 


### PERSONAL.

Dr. Leonard Pearson recently addressed a very large meeting of farmers in Lancaster County, on "Diseases Incidental to Dairy Cows."

Dr. F. B. Grenside, of Guelph, Canada, will remove to New York to take the general management of Mr. S. H. Harland's Belmont Stud.

The many friends of Veterinarian S. E. Weber will be glad to learn that he is slowly recovering from the very serious illness which has afflicted him for the past year.

Dr. J. C. Shaub, of Lancaster, was recently seriously injured by one of his equine patients striking him with his forefeet and kicking him after he was knocked down.

Dr. G. F. Harker, of Trenton, New Jersey, has been sent to investigate the extent and direct the control of an outbreak of hog-cholera in the western part of New Jersey.

Dr. W. Runge, of Newark, New Jersey, has been sent by the State Board of Health into the district around Newport, where anthrax has prevailed for the past month.

Dr. John M. Parker, of Haverhill, Massachusetts, President of the Massachusetts State Veterinary Medical Association, has been at the Pasteur Institute, New York City, undergoing treatment as a safeguard against the effects of a bite.

Dr. J. C. McNeil has been appointed City Veterinarian for Pittsburg, a position now of much importance in that city, as it places the selection and sale of all horses under his direction, thus concentrating responsibility as to the proper selection as well as the general condition of the same on the veterinarian.

Dr. D. McEachran, of the Veterinary Department of McGill University, will address the Convention of Veterinary Faculties of North America at Des Moines on the topic of a "Uniform Title for Veterinarians." We are glad to know that we are going to be favored with the presence of Dr. McEachran at the meeting of the U.S.V.M.A.

Prof. John W. Adams will prepare a paper for the coming meeting of the Pennsylvania State Veterinary Medical Association at their meeting at Cresson Springs on September 3, 1895.

### MISSOURI STATE VETERINARY MEDICAL ASSOCIATION.

THE annual meeting will be held at Moberly, September 4, 1895. Among others the following papers will be presented for discussion: "Tetanus in the Ox," by Dr. T. B. Howard; "Points in Practice," by Dr. J. H. Wattles; "Corn-disease," by Dr. L. E. Booth; "Variations of Opinions," by Dr. L. M. Klutts; "The State Sanitary Veterinary Service," by Dr. T. E. White. These will be followed by others of equal interest and importance.

The convention will be held in the City Hall, and the members will be welcomed by Mayor Willard Cave, who, will be responded to by Dr. Wolf, of Kansas City, or Dr. Ellis, of St. Louis, and, after the routine work, the papers will be read.

The Secretary, Dr. H. B. Piatt, St. Louis, says in his announcement: "At the meeting some one will lead the discussion on each subject. We would ask your assistance in this work, thereby enabling us to keep in good fellowship, brighten up our corroded memories, renew old ties, and demolish any and all local or general jealousies that may have previously existed, and hope to see you with us."

### PENNSYLVANIA STATE VETERINARY MEDICAL ASSOCIATION.

THE next semi-annual meeting will be held at the Mountain House, Cresson, September 3d, arrangements having been made with Superintendent W. R. Dunham.

The following gentlemen will read papers: Dr. James Waugh, on "Peracentesis Abdominalis;" Dr. Otto Noack, on "Trichinosis;" Dr. W. L. Rhoads, on "Pathogenesis and Development of Diseases;" Dr. Jacob Helmer, on "Physical Diagnosis," and Dr. Charles Bland (title not given.) There will be reports of cases, etc., by other veterinarians.

Trains will leave Philadelphia 7.00 A.M. and 12.25 P.M., and arrive at Cresson 4.26 and 8.28 P.M. Will leave Cresson 1.00 and 8.12 P.M., arriving at Philadelphia 11.15 P.M. and 4.30 A.M. Round-trip tickets from Philadelphia to Cresson, good until October 31st, are sold at $12.55.

About twenty members have responded to notices sent out. A very fair attendance for a midsummer meeting is expected.

---

### NEW YORK STATE VETERINARY MEDICAL ASSOCIATION.

THE annual meeting will be held at the New York College of Veterinary Surgeons, New York City, on the 5th and 6th of September next.

Among the important papers to be read will be one by Dr. Claude D. Morris, of Pawling, N. Y., on "Veterinary Sanitation;" Dr. Anderson Crowforth, of Lockport, N. Y., on "Acarus Demolex Folliculorum." Among those who are expected to read, but whose subjects have not been announced at this writing, are Drs. John Wende, Bell, L. E. Willyoung, and Professor Law.

A part of one day will be devoted to the performance of several operations. Arytenectomy will be performed by Professor S. J. J. Harger, of Philadelphia.

---

### IOWA STATE VETERINARY MEETING.

THE eight annual meeting will be held at the Savery Hotel, Des Moines, on September 9th. The Secretary has sent out a very attractive prospectus for this meeting, and encloses a strong and cordial invitation to attend the U. S. V. M. A., which follows the State meeting on the 10th, 11th, and 12th.

Protective veterinary legislation will be among the subjects discussed, and better sanitary laws from a board of health point of view will be considered. After very important committee reports, papers will be read by Drs. S. H. Kingery, of Creston, Iowa, and W. L. Williams, of Bozeman, Montana. Dr. J. C. Schrader, President of the State Board of Health, will lead the discussion on sanitary legislation. A very large attendance is expected.

### WISCONSIN SOCIETY OF VETERINARY GRADUATES.

THE meeting of this Society that was announced for August 14th, at Chippewa Falls, was countermanded, and no meeting will be held until the annual meeting in February, 1896. The general lack of professional prosperity and the expense of members, incidental to the meeting, are given as influencing causes for this decision.

---

THE CALIFORNIA STATE VETERINARY MEDICAL ASSOCIATION will meet on September 10, 1895, at Sacremento, and it will be in order for this Association and the national organization to exchange greetings. We had hoped to have a copy of the programme to announce with this number, but we are forced to go to press without it.

